# Doxycycline hinders phenylalanine fibril assemblies revealing a potential novel therapeutic approach in phenylketonuria

**DOI:** 10.1038/srep15902

**Published:** 2015-10-29

**Authors:** Ada De Luigi, Alessandro Mariani, Massimiliano De Paola, Andrea Re Depaolini, Laura Colombo, Luca Russo, Valeria Rondelli, Paola Brocca, Lihi Adler-Abramovich, Ehud Gazit, Elena Del Favero, Laura Cantù, Mario Salmona

**Affiliations:** 1 Department of Molecular Biochemistry and Pharmacology, Via Giuseppe La Masa 19, Milano, 20156, Italy; 2 Department of Environmental Health Sciences, IRCCS-Istituto di Ricerche Farmacologiche “Mario Negri”, Milano, 20156, Italy; 3 Department of Medical Biotechnology and Translational Medicine, University of Milan, LITA, Segrate, 20090, Italy; 4 Department of Molecular Microbiology and Biotechnology, George S. Wise Faculty of Life Sciences; 5 Department of Oral Biology, The Goldschleger School of Dental Medicine, Tel Aviv University, Tel Aviv 69978, Israel

## Abstract

A new paradigm for the aetiopathology of phenylketonuria suggests the presence of amyloid-like assemblies in the brains of transgenic mouse models and patients with phenylketonuria, possibly shedding light on the selective cognitive deficit associated with this disease. Paralleling the amyloidogenic route that identifies different stages of peptide aggregation, corresponding to different levels of toxicity, we experimentally address for the first time, the physico-chemical properties of phenylalanine aggregates via Small Angle, Wide Angle X-ray Scattering and Atomic Force Microscopy. Results are consistent with the presence of well-structured, aligned fibres generated by milliMolar concentrations of phenylalanine. Moreover, the amyloid-modulating doxycycline agent affects the local structure of phenylalanine aggregates, preventing the formation of well-ordered crystalline structures. Phenylalanine assemblies prove toxic *in vitro* to immortalized cell lines and primary neuronal cells. Furthermore, these assemblies also cause dendritic sprouting alterations and synaptic protein impairment in neurons. Doxycycline counteracts these toxic effects, suggesting an approach for the development of future innovative non-dietary preventive therapies.

Mutations in the phenylalanine hydroxylase (PAH) gene cause the partial or total inhibition of enzyme activity^1^. An excess of phenylalanine (Phe), resulting from failure in the biotransformation to tyrosine, is at the basis of the onset and progression of phenylketonuria (PKU)^2^. PAH is expressed not only in the liver but also in the kidneys, pancreas and brain^3^, and deficiency in the conversion of Phe may be mild (in the case of hyperphenylalaninemia (HPA)) or moderate to severe (in the case of PKU) in association with poorly tolerated (360–600 μmol/L) or toxic (>600 μmol/L) plasma levels in a free diet condition. High and persistent levels of Phe are teratogenic during foetal development (microcephaly, congenital heart defects), and continue after birth if a controlled diet is not followed. Notably, in the 1950s it was demonstrated that high levels of blood Phe also cause neuropsychological deficiencies^4^. Pharmacological intervention as a non-dietary alternative to prevent or mitigate the cognitive deficit associated with PKU is increasingly desirable. The development of a therapeutic strategy extending to all PKU patients requires an understanding of the molecular basis of the deficit progression, i.e., the mechanism of the disease. The connection between metabolite accumulation and neuronal cell malfunction is still unclear, even though several mechanisms have been proposed to explain the neuropathological findings observed in PKU. Recently, a new paradigm has been put forward to explain the aetiopathology of PKU^5^. Attention has been drawn to the features of the Phe precipitate that forms above the solubility limit. Long uniaxial aggregates, indicated as amyloid-like structures, have been observed in aqueous solutions both *in vitro* and in transgenic mice and PKU patients. These have been proven to display cytotoxicity that can be neutralized by specific antibodies. Although quite different from the typical amyloid fibres, nonetheless this finding is suggestive of a novel approach to the molecular basis of PKU, addressing the possible role of Phe aggregates in the disease, interfering with the cellular functions. The propensity to form fibrils depends on the concentration of Phe in the milliMolar range that promotes a tight packing of the aromatic ring in ordered structures (hydrophobic π-π stacking interaction)^6^,^7^. The fibrillar morphology, as defined by the characteristic birefringence under cross-polarization, has also been confirmed by light scattering, thioflavin T and ANS binding assays^8^. It has been suggested that aromatic interaction plays a crucial role in creating an ordered configuration and energetic contribution in the molecular assembly of amyloid structures. Notably, in amyloidogenic peptides the substitution of the aromatic amino acid is crucial to the fibrillation process; therefore, the study of the self-assembly of Phe fibrils offers the opportunity to discover antibodies or small molecules for a novel therapeutic approach^9^.

In this paper, paralleling the amyloidogenic route that identifies different stages of peptide aggregation, corresponding to different levels of toxicity, we experimentally address the physico-chemical properties of Phe aggregates before confirming that anti-Phe assembly antibodies counteract their toxicity, as determined in primary neuronal cells and immortalized cells. This strategy was pursued to unequivocally determine that Phe assemblies are not only supermolecular structures, but also have a biological activity that can be a target for pharmacological therapy.

In the light of these observations, in an attempt to identify a novel pharmacological approach for PKU^10^ we have focused our attention on doxycycline (Doxy) for its capacity to hinder amyloidogenic peptides and protein aggregation, destabilize protein aggregates and promote their degradation by proteases^11^,^12^,^13^,^14^.

In the last fifteen years, following our first report showing that tetracyclines inhibited the aggregation of prion protein fragments and Alzheimer’s β peptides, a large amount of research has showed that tetracyclines inhibit the aggregation of transthyretin, W7FW14F apomyoglobin, amylin, huntingtin, α-synuclein, alpha-macroglobulin, polyadenylate binding nuclear protein 1 and immunoglobulin light-chain^15^,^16^,^17^. The blood brain barrier passage of Doxy was recently confirmed in patients suffering from Creutzfeldt-Jakob disease, since its concentrations could be determined in autopsy samples treated with 100 mg Doxy daily. Levels ranging between 646–3,051 ng/g of brain tissue were measured in individuals who received the last dose within 24 h of death^16^. The use of Doxy as an orphan drug for the treatment of systemic amyloidosis caused by mutated transthyretin (Treatment of familial amyloid polyneuropathy. EU/3/12/955) and of β2-microglobulin amyloidosis (Treatment of systemic amyloidosis caused by β2-microglobulin. EU/3/12/961) was recently approved by the European Committee for Orphan Medicinal Products. Tetracyclines are involved in more than 130 clinical trials, unrelated to their antimicrobial activity^18^.

## Results

### Small angle X-ray scattering on deposited phenylalanine aggregates

X-ray scattering experiments were performed to obtain structural information about the supramolecular organization of Phe aggregates and reveal the effect of the presence of Doxy on their structure. Solutions, at a final concentration of 20 mM in ddH_2_O, were prepared and left to dry on a thin kapton support in the presence of a 7T magnetic field. This procedure has been already applied to induce macroscopic alignment of fibers of small amyloidogenic peptides during slow evaporation of aqueous solvent^19^,^20^.

**Figure 1:**
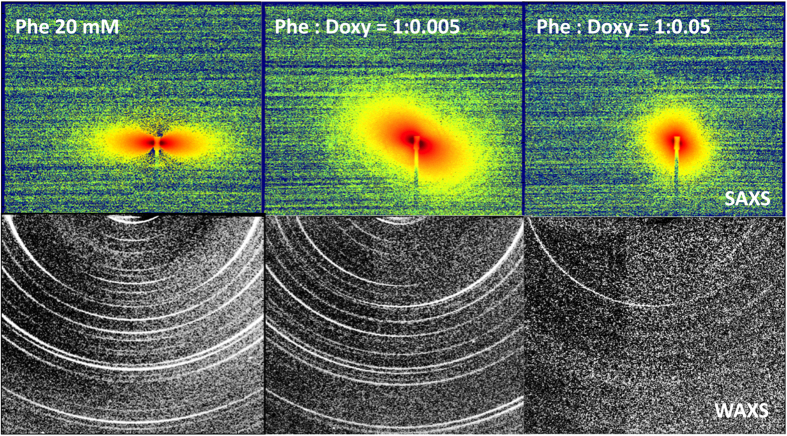
X-ray scattering 2D patterns for pure 20 mM Phe (left column) and mixed Phe:Doxy, 1:0.005 (central column) and 1:0.05 molar ratios (right column). Solutions were prepared in ddH_2_O and allowed to dry on a thin Kapton support. Images show SAXS (top row) and WAXS (bottom row) patterns.

Figure 1 shows the 2D images, in the SAXS and WAXS regions (top and bottom rows, respectively), collected for pure Phe and mixed Phe:Doxy (1:0.005 and 1:0.05 molar ratios, simultaneously dissolved). The SAXS intensity patterns are very different. The Phe scattered pattern (top row, left panel) is typical for long fibres aligned in the direction of the vertical axis. The macroscopic organization of fibres is not determined by the applied external magnetic field, as different regions of the sample show different orientations. Alignment instead seems to be governed by dewetting and dehydration; however, in each region, it extends on a length scale comparable to the X-ray beam cross size (0.3 × 0.2 mM). Even at a low molar ratio, the presence of Doxy dramatically affects the SAXS diffraction pattern (Phe:Doxy = 1:0.005), as visible in Fig. 1 (top row, centre panel). A long-range supramolecular organization of Phe after dehydration is still observable, giving rise to the small-angle scattered intensity, but alignment of fibres is strongly reduced. For higher Doxy content (Phe:Doxy = 1:0.05; top row, right panel), the SAXS pattern is almost isotropic and the total scattered intensity is lower, as for less structured systems.

On the other hand, X-ray scattering experiments in the WAXS region allow the investigation of the supramolecular organization of the different systems at a local scale (<1 nm). The intensity patterns are reported in Fig. 1, bottom row. In the case of pure Phe (left panel), intensity maxima are arcs centred on meridional or equatorial axes, thus pertaining to along or across organization with respect to the elongation / alignment axis of the aggregated structures. The regrouped Phe spectrum, with the angular position of all peaks, is reported in the Supplementary Information (Figure S1). Results are consistent with the presence of well-structured, aligned fibres. Our findings are in good agreement with recent results obtained by molecular dynamics simulation studies^6^ and X-ray crystallography^7^. We observe equatorial peaks in the 5Å-range, consistent with the proposed repetition distance of piled Phe rings, while a leading peak is seen on the 15Å-range distances, consistent with the proposed lateral organization of Phe strings in a thicker fibre. Besides differences in the details of local arrangement, twofold or fourfold structures at the molecular level, both previous studies identify hydrogen bonding and π-π stacking as the dominant short range interactions that scale up to give rise to axially ordered aggregates. Our results show that the presence of Doxy affects the local structure of Phe aggregates, preventing the formation of well-ordered crystalline structures, as shown in Fig. 1 (right panel). In particular, as evidenced in the Supplementary Information, the presence of small amounts of Doxy hinders the lateral organization, as indicated by an important depletion of the corresponding order peak (at q = 4.4 nm^−1^) and by the inversion of the relative weight of the meridional and equatorial peaks intensities. This suggests that, at this Doxy content, individual Phe piling up is still preserved at the expense of the lateral organization of Phe strings, maybe enhancing their chimeric behaviour. At higher Doxy content structuring is hindered at all lenghtscales.

### Atomic force microscopy of phenylalanine assemblies: interference of doxycycline

AFM studies were carried out to clarify the morphology of Phe assemblies prepared in different solvents with different pH and salt conditions, in the absence or in the presence of Doxy.

**Figure 2:**
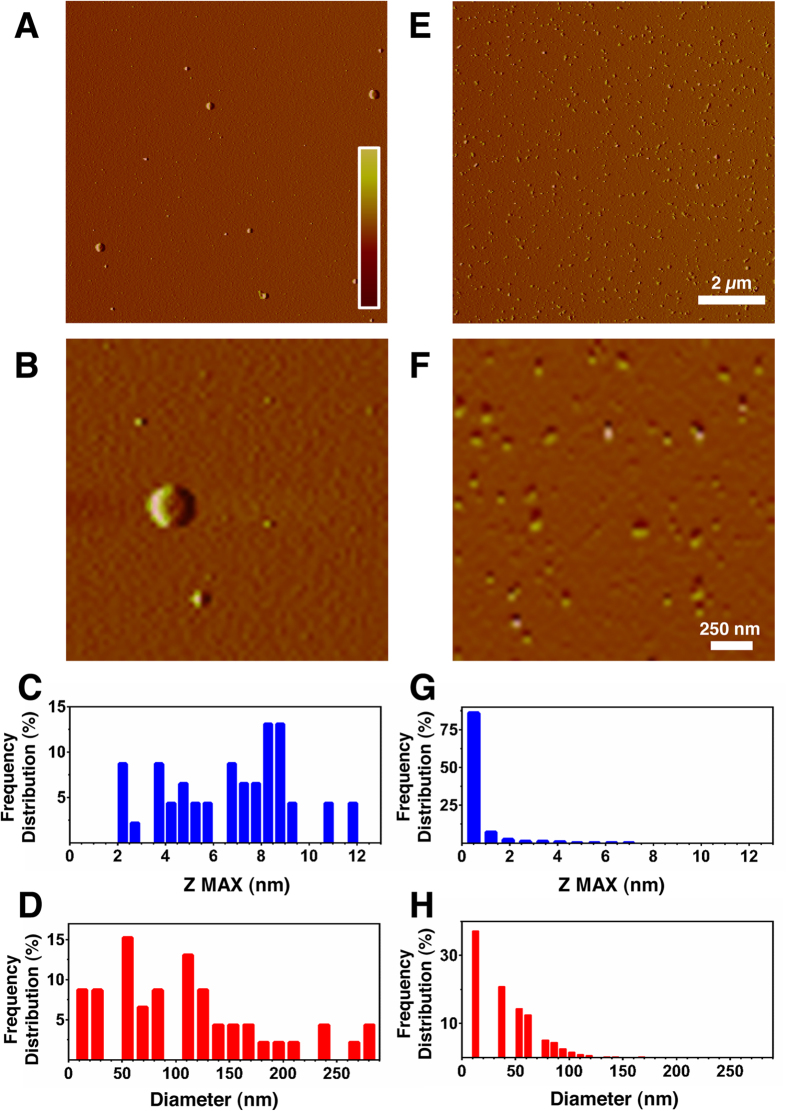
Tapping mode atomic force microscopy images (amplitude data). Images (Panels (**A**,**B**) refer to pure Phe and co-dissolved Phe:Doxy at 1:0.005 molar ratio (**E**,**F**), freshly prepared in ddH_2_O. Diameters and heights were determined by SPIP analysis. Panels (**C**,**D**,**G**,**H**) show the frequency distribution of the different assemblies obtained for the two systems. The scale bars correspond to an amplitude range of −25/ + 25 mV for panels (**A**,**E**) and −10/ + 10 mV for panels (**B**,**F**).

Figure 2 shows images relative to 1 mM Phe (left column) and Phe:Doxy (1:0.005) (right column) in ddH_2_O. It can easily be seen that the aggregative behaviour of the two systems is completely different, the one containing Doxy being populated by smaller aggregated structures. In the case of the pure Phe sample, a broad distribution of species is seen, ranging from 2 to 12 nm in height (Fig. 2A) and from 10 to 290 nm in diameter (Fig. 2B), with two major populations in the 50–90 nm (31%) and 100–220 nm (41%) ranges (Fig. 2B). The co-incubation with Doxy (1:0.005 molar ratio) had a remarkable influence on the height and diameter of the observed species (Fig. 2D,E). In particular, 98% of them were under 5 nm in height (Fig. 2G) and under 100 nm in diameter (Fig. 2H).

The same effect of Doxy in hindering Phe aggregation has been observed at different pH values, namely pH 4.7 and pH 9.2, as reported in Figures S2 and S3 of the Supporting Information.

**Figure 3:**
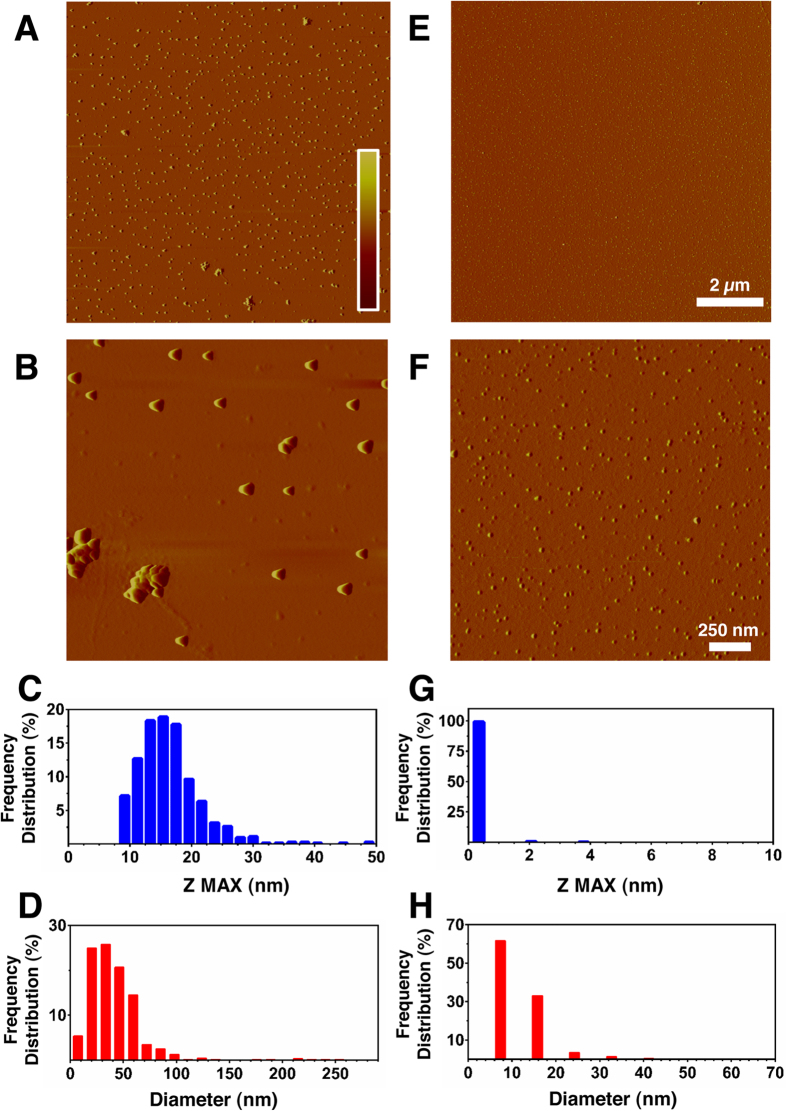
Tapping mode atomic force microscopy images (amplitude data). Images (Panels (**A**,**B**) refer to pure Phe and co-dissolved Phe:Doxy at 1:0.005 molar ratio (**E**,**F**), freshly prepared in PBS (10 mM, pH 7.4). Diameters and heights were determined by SPIP analysis. Panels (**C**,**D**,**G**,**H**) show the frequency distribution of the different assemblies obtained for the two systems. The scale bars correspond to an amplitude range of −25/ + 25 mV for panels (**A**,**E**) and −10/ + 10 mV for panels (**B**,**F**).

In order to mimic the experimental conditions of studies carried out on primary neurons and immortalized cell lines and check the effect of the presence of salt, AFM measurements were also performed in 10 mM PBS, pH 7.4. Figure 3 shows images relative to 1 mM Phe (left column) and Phe:Doxy (1:0.005) (right column) in PBS. Again, the aggregative behaviour of the two systems is completely different, the one containing Doxy being populated by smaller aggregated structures. In the case of the pure Phe sample, a broad distribution of species is observed, ranging from 10 to 30 nm in height (Fig. 3C) and from 25 to 100 nm in diameter (Fig. 3D). The co-incubation with Doxy (1:0.005 molar ratio) had a remarkable influence on the size of the observed species (Fig. 3E–H), with 98% under 1 nm in height (Fig. 3G) and 65% under 15 nm in diameter (Fig. 3H).

### Phenylalanine assemblies as a target for pharmacological therapy

**Figure 4:**
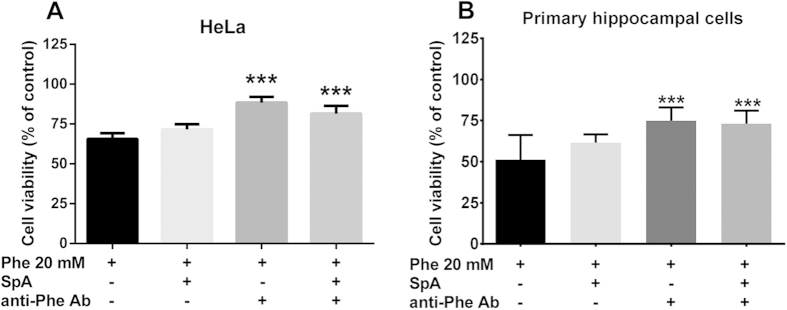
Effect of immunodepletion of phenylalanine assemblies. HeLa cell line (**A**) or primary neuronal cells (**B**) were incubated with 20 mM Phe solutions for 24 or 72 h, respectively, after immunoprecipitation of Phe assemblies with anti-Phe antibody (anti-Phe Ab 1:10) or after immunodepletion using Sepharose-Protein A beads (SpA). Cell viability was determined using the MTT or MTS assay. Values are mean ± standard deviation of 9 samples obtained in 3 different experiments. ***p < 0.001 vs Phe (20 mM). One-way ANOVA followed by Dunnett’s multiple comparisons test.

Before carrying out *in vitro* toxicity tests on immortalized and primary neuronal cells, we tested the cytotoxicity of Phe assemblies alone and confirmed that antibodies raised against them could hinder or counteract this effect. To this end, 20 mM Phe were dissolved in culture medium, anti-Phe antibody preparation was added to the culture medium to immunoprecipitate the aggregates and cell toxicity studies were carried out. As a supplementary approach to remove Phe assemblies, a separate batch of the medium was also incubated with Sepharose-Protein A beads to deplete all immunoglobulins present in the medium. HeLa cells treated with a solution containing 20 mM Phe assemblies showed 65.7 ± 3.5% viability; after immunoprecipitation the viability of cells was increased to 88.3 ± 3.7%, determined via MTT assay. Moreover, the total depletion of the immune complex from the culture medium using Sepharose-Protein A beads increased cell viability to 81.5 ± 4.9%, confirming the selectivity of the antibody against Phe assemblies (Fig. 4A). As supplementary controls, we also tested the effect on cell viability (in brackets) of: i) 20 mM Phe assemblies incubated with Sepharose-Protein A beads alone (71.7 ± 3.2%), ii) control rabbit IgG (66 ± 9.5%), iii) Sepharose-Protein A beads alone in the absence of Phe (108 ± 15.8%) and iv) anti-Phe antibody alone (93.5 ± 11.9%).

Incubation with Sepharose-Protein A beads and anti-Phe antibody were used as negative controls for Phe effects in hippocampal mixed cultures, as described above for HeLa cells. The reduction of neuronal viability by 20 mM Phe (49.7 ± 14.9%) was also significantly increased to 74.9 ± 8.2% in the presence of anti-Phe antibody (*p* < 0.001 vs Phe). No effects of Sepharose-Protein A beads incubation alone were observed (Fig. 4B).

### Toxicity of phenylalanine assemblies on SH-SY5Y and HeLa cell lines

**Figure 5:**
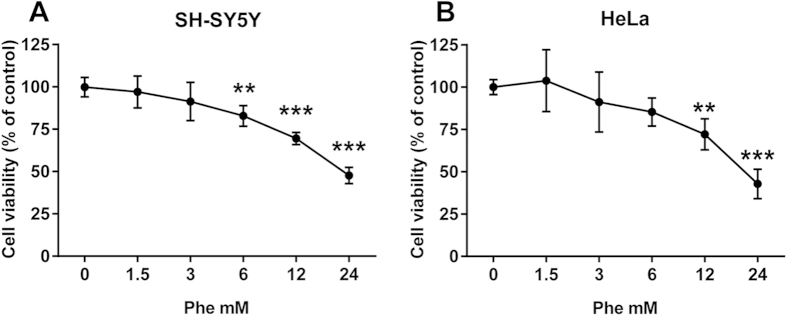
Toxicity of phenylalanine assemblies in SH-SY5Y and HeLa cell lines. Phe assembly toxicity was determined via MTT assay after 24 h of incubation in human neuroblastoma SH-SY5Y (**A**) and human adenocarcinoma HeLa (**B**) cell lines. Values are mean ± standard deviation of 9 samples obtained in 3 different experiments. **p < 0.01, ***p < 0.001 vs control. One-way ANOVA followed by Dunnett’s multiple comparisons test.

In neuronal and non-neuronal human cell lines the growth inhibition after high concentration of Phe was dose-dependent (Fig. 5A,B). The SH-SY5Y cell line showed a statistically significant reduction in viability starting from 6 mM Phe, after 24 h of treatment (−17.2%; 95% CI 6.498 to 27.84) that was more pronounced at 12 and 24 mM (−31% and −52%; 95% CI 19.83 to 41.17 and 41.66 to 63.00, respectively). HeLa cell viability was affected by the treatment at 12 mM of Phe (−28%; 95% CI 9.301 to 46.37) and became more pronounced at 24 mM (−57%; 95% CI 38.63 to 75.70). Similar EC50 values were obtained for both cell lines (SH-SY5Y 22.6 mM, 95% CI 19.2 to 26.6 mM; HeLa 20.5 mM, 95% CI 16.6 to 25.3 mM). The incubation of both cell lines with 12 mM Ala (used as a negative control) for 24 h did not influence cell viability (SH-SY5Y 101 ± 16%; HeLa 102 ± 7.5%).

### Reversal of phenylalanine assemblies toxicity by doxycycline

**Figure 6:**
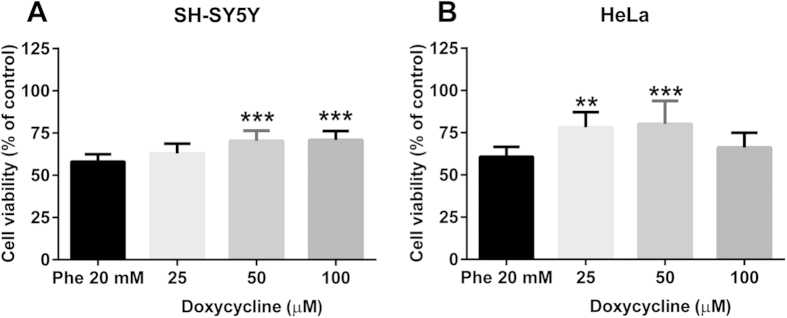
Effect of doxycycline on phenylalanine assemblies toxicity. SH-SY5Y (**A**) and HeLa (**B**) cell lines were treated with 20 mM of Phe alone or after co-incubation with 25–50–100 μM of Doxy for 24 h. Cell viability was assessed by MTT assay. Values are mean ± SD of 12 samples from 4 different experiments. **p < 0.01, ***p < 0.001 vs Phe alone. One-way ANOVA followed by Dunnett’s multiple comparisons test.

After establishing the most appropriate experimental conditions, we tested whether doxycycline could reduce Phe assembly toxicity. Three Phe:Doxy molar ratios were used in all experiments: 20 mM:100 μM, 20 mM:50 μM and 20 mM:25 μM. In the SH-SY5Y cell line (Fig. 6A), Doxy was effective down to 50 μM, increasing cell viability from 58 ± 4.3% to 70 ± 6.1%. A similar increase was observed with 100 μM (71 ± 5.4%). In the HeLa cell line (Fig. 6B), Doxy was effective at 25 μM and 50 μM, increasing viability from 61 ± 13.7% to 78 ± 9.0% and 80 ± 13.7%, respectively. As a negative control we used the antibiotic gentamicin, which is structurally unrelated to Doxy. Under our experimental conditions, this drug was unable to counteract Phe assemblies toxicity (data not shown). The cell viability after treatment with 100 μM Doxy alone was unaffected in both cell lines (SH-SY5Y: Doxy 96 ± 15.8%; HeLa Doxy 106 ± 16.9%).

### Inhibition of phenylalanine hydroxylase activity by alpha methyl phenylamine. Effect of doxycycline

**Figure 7:**
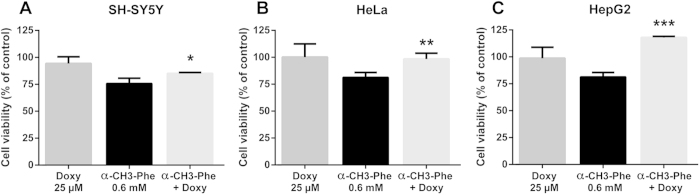
Inhibition of phenylalanine hydroxylase by alpha methyl phenethylamine. Effect of doxycycline. SH-SY5Y(**A**), HeLa (**B**) and HepG2 (**C**) cell lines were incubated with 0.6 mM of α-methyl-Phe alone or after co-incubation with 25 μM of Doxy for 24 h. Cell viability was assessed by MTT assay. Values are mean ± standard deviation of 9 samples from 3 different experiments. *p < 0.05, **p < 0.01, ***p < 0.001 vs α-methyl-Phe alone. One-way ANOVA followed by Dunnett’s multiple comparisons test.

The chemically induced model of hyperphenylalaninemia is an alternative experimental approach for mimicking the pathological condition of Phe accumulation. To carry out this study we also used a HepG2 cell line of hepatic origin that is known to express phenylalanine hydroxylase activity. All three cell lines treated with 0.6 mM α-methyl-Phe for 24 h showed a significant reduction in cell viability to 76 ± 5.0%, 81 ± 4.5% and 81 ± 4.2% in SH-SY5Y (A), HeLa (B) and HepG2 (C) cell lines. Co-treatment with 25 μM Doxy had a differential protective effect on all three cell lines, increasing cell viability (SH-SY5Y: 85 ± 1.0%, HeLa: 98 ± 5.5%, HepG2 118 ± 1.0, Fig. 7).

### Neurotoxicity of phenylalanine assemblies

**Figure 8:**
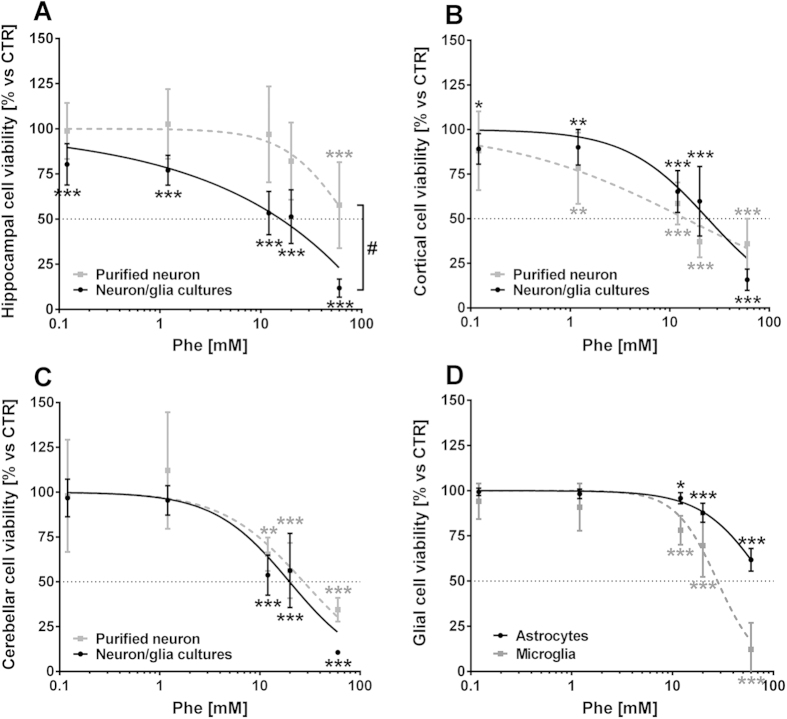
Phenylalanine effects on primary neuron cultures. Purified or mixed neuron/glia cultures were obtained from different CNS areas of mouse embryos (E13), and treated with Phe (from 0.12 up to 60 mM) for 72 h. (**A**–**C**) *Solid lines*—Phe dose-response curve in mixed neuron/glia cultures. Phe EC50 (CI95): 14.26 mM (11.24–18.1) in hippocampal cultures; 19.78 mM (16.47–23.75) in cerebellar cultures; 24.12 mM (20.56–28.30) in cortical cultures. *Dotted lines*—Phe dose-response curve in purified neuron cultures. Phe EC50: 73.58 mM (47.85–113.1) in hippocampal cultures; 27.34 mM (18.09–41.3) in cerebellar cultures; 14.42 mM (9.39–22.13) in cortical neuron cultures. (**D**) *Solid line*—Phe dose-response curve in purified astrocyte cultures. Phe EC50: 84.25 mM (75.84–93.60). *Dotted line*—Phe dose-response curve in purified microglia cultures. Phe EC50: 27.93 mM (23.66–32.96). *p < 0.05, **p < 0.01, ***p < 0.001 versus control. One-way ANOVA and Dunnett’s post-test. ^#^*p* < 0.05 mixed neuron/glia cultures vs purified neuron cultures. Paired *t*-test.

The effect of Phe on neuron and glia viability was investigated in mixed neuron/glia and purified neuron cultures obtained from different brain areas. After six days, cultures were treated for 72 h with different concentrations of Phe (from 0.12 to 60 mM). Dose-response curves carried out on mixed neuron/glia cultures showed that distinct brain areas have different sensitivity to Phe toxicity, with the hippocampus in particular showing higher vulnerability (EC50: 14.26 mM) than cerebellar (19.78 mM) or cortical (24.12 mM) cultures (Fig. 8A–C, solid lines). Phe significantly reduced neuron viability by 0.12 mM in hippocampal (80.3 ± 11.5%, *p* < 0.001 vs CTR) and cortical cultures (89.2 ± 8.6%, *p* < 0.05 vs CTR), but not in cerebellar cultures. The lowest Phe effective concentration in the latter was 12 mM, which reduced cell viability to 53.8 ± 11.2% (*p* < 0.001 vs CTR). Treatment with alanine (up to 20 mM) did not affect neuron/glia viability in any type of culture (data not shown).

To investigate the cell-specific effects of Phe, purified cell cultures (neurons or glia) were used. A dose-dependent toxicity of Phe was shown for purified neurons, with higher sensitivity for cortical (EC50: 14.42 mM) and cerebellar (27.34 mM) neurons compared to hippocampal neurons (73.58 mM; Fig. 8A–C, dotted lines). Interestingly, purified neurons from the hippocampus showed a lower vulnerability to Phe treatment as compared to mixed neuron/glia cultures from the same brain area (*p* < 0.05, Paired *t*-test for the dose-response curves). In fact, Phe only induced significant reduction in purified hippocampal neuron viability from 60 mM (57.8 ± 23.8%, *p* < 0.001 vs CTR; Fig. 8A).

For glial cells, microglia (EC50: 27.93 mM) were more sensitive than astrocytes (84.25 mM) to Phe cytotoxicity (Fig. 8D). A significant decrease in cell viability was observed at doses as low as 12 mM Phe, both in microglia (78.1 ± 8%, *p* < 0.001) and in astrocyte cultures (95.8 ± 3.1%, *p* < 0.05 vs CTR).

### The cell death mediated by phenylalanine assemblies is counteracted by doxycycline

**Figure 9:**
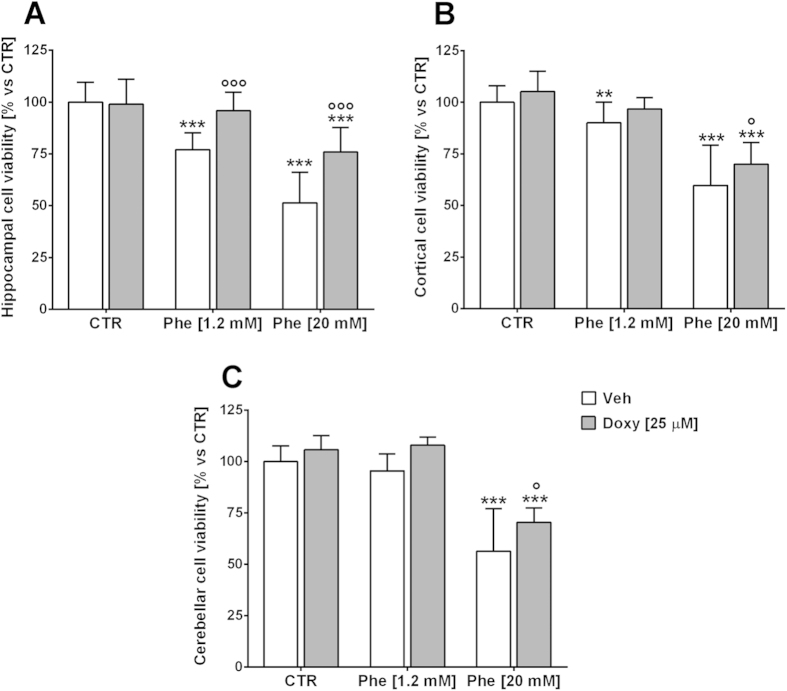
Doxycycline reduces phenylalanine toxicity in neuron/glia cultures. Mixed neuron/glia cultures from different CNS areas were exposed to 1.2–20 mM Phe alone or in co-treatment with 25 μM Doxy, for 72 h. Protective effects by Doxy were shown in cultures from the hippocampus, where both 1.2 and 20 mM Phe toxicity were significantly counteracted (**A**). A significant decrease in the cell death induced by 20 mM Phe was also observed in cortical (**B**) and cerebellar (**C**) cultures. **p < 0.01, ***p < 0.001 versus control. °*p* < 0.05, °°°*p* < 0.001 vs Phe. F (DFn, DFd) interaction: hippocampal cultures F (2, 200) = 17.15, *p* < 0.001; cerebellar cultures F (2, 89) = 0.7708, *p* = 0.4657; cortical cell cultures F (2, 171) = 0.5546, *p* = 0.5753. Two-way ANOVA and Bonferroni’s post-test.

Co-treatments with Doxy were performed in mixed neuron/glia cultures to investigate whether Doxy was able to prevent Phe-induced neuron death. When mixed cultures were co-treated with 25 μM Doxy, the 20 mM Phe-dependent reduction of cell viability was significantly counteracted in hippocampal (from 51.3 ± 14.8% to 75.9 ± 11.9%; *p* < 0.001), cerebellar (from 56.7 ± 20.7% to 70.4 ± 7%; *p* < 0.05) and cortical cultures (from 59.8 ± 19.5% to 70 ± 10.4%; *p* < 0.05; Fig. 9A–C). A protective effect of Doxy was also observed for the cell toxicity induced by 1.2 mM Phe, although this was only seen in the hippocampal cultures (*p* < 0.001; Fig. 9A), probably due to the higher Phe toxicity observed in these cultures.

### Prevention of phenylalanine assemblies dependent dendritic sprouting alterations and synaptic protein impairments by doxycycline

The effects of Phe (1.2–20 mM) on dendritic arborisation and synaptic protein expression were analysed in a hippocampal “sandwich” neuron/glia model. After 10 days, co-cultures were treated for 72 h and immunostained for mature neuronal filament markers (NF200) and the presynaptic protein synaptophysin. Dendrite outgrowth evaluation and semiquantification of the synaptophysin signal were performed on 3D reconstructed neuritis for each different treatment condition.

**Figure 10:**
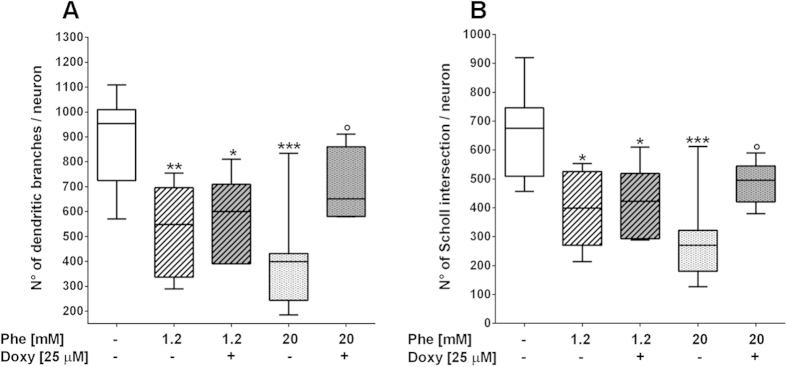
Phenylalanine-induced dendritic sprouting alterations in hippocampal neurons prevented by doxycycline co-treatment. Hippocampal “sandwich” co-cultures were treated with Phe/Doxy for 72 h. The neuron fractions were stained with NF200 and nuclei were marked by Hoechst 33258. At least eight fields (600x) for each condition from three independent experiments were analysed. The number of dendritic branches (**A**) and Sholl intersections (**B**) was significantly decreased by Phe treatments. Treatment with 25 μM Doxy significantly counteracted the dendritic alterations induced by 20 mM Phe. *p < 0.05, **p < 0.01, ***p < 0.001 versus control. °*p* < 0.05 vs 20 mM Phe. One-way ANOVA and Tukey’s post-test.

**Figure 11:**
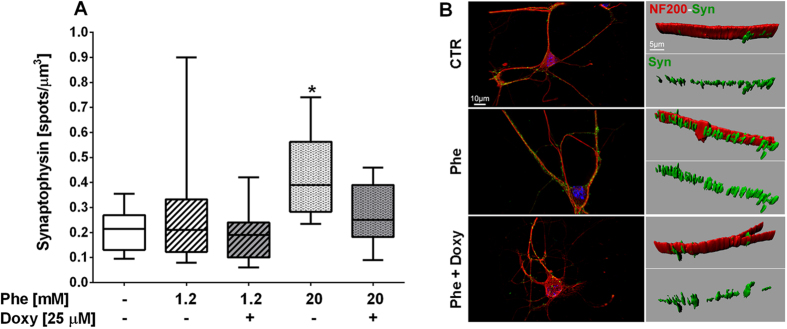
Synaptophysin density alterations in phenylalanine-treated hippocampal neurons. Hippocampal “sandwich” co-cultures were treated with Phe/Doxy for 72 h. Cells were double-stained with NF200 (red) and synaptophysin (green); nuclei were marked by Hoechst 33258 (blue). The number of synaptophysin-positive spots was normalized to the total volume of neurofilaments (NF200 expression) after three-dimensional reconstruction of the marker signals (represenative images in (**B**). At least ten fields (1200x) for each condition from three independent experiments were analyzed. (**A**) The density of synaptophysin-positive spots was significantly increased by 20 mM Phe (*p < 0.05 versus control; One-way ANOVA and Dunnett’s post-test). Treatment with 25 μM Doxy showed a trend towards a reduction in synaptophysin density, increasing towards control levels.

A significant decrease in the number of dendritic branches and Sholl intersections (specific analysis for branching complexity) was induced by Phe compared to the control condition by about 40–55% (**p < 0.01 and ***p < 0.001 versus control for 1.2 or 20 mM Phe, respectively; Fig. 10A,B). Co-treatments with 25 μM Doxy partially prevented the 20 mM Phe-induced alterations in dendrite outgrowth (*p* < 0.05; Fig. 11A,B). The density of synaptophysin-positive spots was significantly increased by 20 mM Phe treatment (*p < 0.05 versus control; Fig. 11 A and representative images in 11 B). Co-treatment with 25 μM Doxy showed a trend to reduce the synaptic alteration induced by Phe towards control levels.

## Discussion

An early study^5^ indicated for the first time the presence of amyloid-like assemblies in the brains of transgenic mouse models and patients with PKU. The co-localization of typical hallmarks of amyloid-type structures and the presence of Phe (Congo red staining and anti-Phe immunostaining) was used as an indication that self-assembled structures of Phe could accumulate in the brain and potentially cause neurological illness. As a proof of Phe assemblies toxicity, the specificity and efficacy of the corresponding antibodies was established via immune-gold electron microscopy and the reduction of their cytotoxicity in Chinese hamster ovary cell lines.

On the other hand, the supramolecular organization of Phe has been observed and studied by X-ray crystallography^7^, following the claimed role of aromatic interaction in building ordered configurations and providing energetic contribution to the molecular assembly of peptides in amyloid structures^9^. In fact, Phe assemblies have been found to assume a supramolecular strand-like structure^21^ where adjacent layers disposition is seemingly stabilized by parallel-displaced π-π interactions between the aromatic moieties. Edge-to-face π stacking interactions are found between the layers and hydrogen bonds between carboxylate and amino groups, stabilizing the non-covalent interactions^7^.

The aim of our study was to explore the molecular basis of PKU, using a novel approach addressing the role of Phe aggregates. In this perspective, the effect of an interfering molecule – Doxy – on both Phe aggregation and Phe cytotoxicity was also examined, possibly helping in the development of future therapies, and aiding in the investigation of other metabolic diseases. We carried out small angle, wide angle X-ray scattering (SAXS and WAXS) and AFM experiments to obtain structural information on the supramolecular organization of Phe and how it is affected by the presence of Doxy. Results were consistent with the presence of well-structured, aligned fibres in the pure Phe system. The presence of Doxy affects the local structure of Phe aggregates, significantly preventing the formation of well-ordered crystalline structures at very low molar ratios. In particular, the early disappearance of lateral organization, while preserving the Phe-piling structure, suggests a role of Doxy in interfering with inter-string H-bond interactions. Both fiber elongation and thickening would be prevented by induced structural fragility.

AFM observation of Phe assemblies, with and without Doxy, revealed that their morphology is similar in solvents mimicking the X-ray diffraction and cellular models studies, namely ddH_2_O and 10 mM PBS, respectively. In both solvents, the presence of Doxy caused a significant reduction in the size of the assemblies. Similar experiments were also carried out at different pH (10 mM acetate buffer, pH 4.7 and 10 mM TRIS-HCl buffer, pH 9.2). In fact, both the Phe local structure^22^ and Doxy ionization state are pH sensitive. Our results showed that the ability of Doxy to hinder Phe assemblies spans from acidic to basic pH^23^.

The present study confirms that Phe at high concentrations forms molecular assemblies that are recognized by a specific antibody which counteracts the associated toxicity. In light of these observations, we tested the efficacy of Doxy on the toxicity of Phe assemblies in immortalized cell lines and primary neuronal cultures. The main outcome of our observations is that primary and immortalized cell lines of neuronal origin are more susceptible to Phe assembly toxicity than non-neuronal ones. This also holds true for the protective effect displayed by Doxy, which only completely counteracted Phe toxicity in non-neuronal cell lines. This may shed light on the selective cognitive deficit associated with PKU. We also carried out a series of experiments aimed at inhibiting PAH activity in three cell lines; to this end, we also took into account the HepG2 cell line which is of hepatic origin and possesses particularly high levels of PAH. In our opinion, this represents proof of concept for the potential efficacy of Doxy in animal models of PKU.

It has been demonstrated in human and animal brains that the hippocampus and cortex are the areas most involved in PKU^5^,^24^,^25^, characterized by progressive neuronal loss, white matter abnormalities, dendritic retraction or failure of arborisation and reduced synaptic density^26^,^27^. All these neuropathological effects may be secondary to the accumulation of high concentrations of Phe^28^.

In our experimental conditions, Phe assemblies were toxic to neuronal cultures. In mixed neuronal/glia co-cultures that recapitulate the complexity of brain parenchyma, the hippocampus and cerebral cortex both have a similar susceptibility to Phe assemblies, while in the absence of glia only purified cortical neurons showed a similar vulnerability. This suggests a possible contribution of glia cells to Phe-mediated neurotoxicity.

This was also confirmed in the co-treatment with Doxy. Hyppocampal mixed cultures were most responsive to Phe toxicity and to reversion by Doxy, whereas cortical and cerebellar mixed cultures were less influenced by the effect of Doxy. The toxicity of Phe assemblies was also evaluated by the analysis of dendritic sprouting and synaptophysin expression in hippocampal culture. The impairment in arborisation and branched projections was evident after treatment with Phe assemblies. This effect was sustained by a concomitant increase in expression of synaptophysin. These neuronal alterations were partially counteracted by co-treatment with Doxy, confirming that protection was evident only in the presence of high concentrations of Phe, which induce the self-aggregation phenomenon.

In conclusion, the study performed is the paradigm of an innovative repositioning approach of a well known antibiotic for the management of neurological diseases. In an applied perspective, the work is characterized by all the advantages associated with drug repositioning over drug development. One such advantage is represented by the availability of information on the pharmacokinetics and toxicity profiles of the pharmacological agent to be tested. Indeed, this type of information is a prerequisite for any clinical study and it minimizes safety issues, accelerating the process of testing the therapeutic efficacy of the selected drug for the new indication. Drug repositioning strategies are likely to boost the role of non-profit and academic institutions in the development of novel therapeutic approaches, as the process is foreseen to result in a significant reduction of associated costs. Finally, drug repositioning is likely to be of particular importance for rare diseases, as it can be envisaged to fill the gap between needs and costs^29^.

## Methods

### X-ray scattering (SAXS and WAXS)

Twenty mM Phe solutions were prepared in doubly distilled (ddH_2_O) together with solutions at Phe:Doxy = 1:0.005 and Phe:Doxy = 1:0.05 molar ratios. Samples were allowed to dry on a thin Kapton support in the presence of a 7T magnetic field. Small angle (SAXS) and wide angle (WAXS) X-ray scattering experiments were performed at the ID02 beamline at the European Synchrotron Radiation Facility (ESRF, Grenoble), with an incident X-ray wavelength λ = 0.1 nm. Spectra were collected in both the SAXS and WAXS configurations, in a wide region of momentum transfer, *q* (0.02 nm^−1^ ≤ *q*  ≤ 35 nm^−1^), to allow for structure determination on different lengthscales. The exposure time for each measurement was 0.2 s to avoid any radiation damage. Collected images were processed and corrected using the standard ID02 software. The X-ray scattering spectra of the empty cells were measured in both configurations and subtracted.

### Atomic force microscopy

Phe samples were dissolved at 1mM in ddH_2_O (pH 5.5) or in PBS (10 mM, pH 7.4), or in acetate buffer 10 mM (pH 4.7) or in TRIS-HCl buffer 10 mM (pH 9.2), with or without Doxy at 1:0.005 molar ratio, and 50 μL were spotted onto a freshly cleaved Muscovite mica disk and incubated for 1 min. The disk was then washed with ddH_2_O, dried under a gentle nitrogen stream and put into a vacuum dryer for 48 h.

Samples were mounted onto a Multimode AFM with a NanoScope V system (Veeco/Digital Instruments) operating in Tapping Mode using standard antimony(n)-doped Si probes (T: 3.5–4.5 mm, L: 115–135 mm, W: 30–40 mm, f_0_: 313–370 kHz, k: 20–80 N/m) (Bruker).

Samples were analyzed with the Scanning Probe Image Processor (SPIP Version 5.1.6 (released April 13, 2011) data analysis package. SPIP software was used to analyse the distribution of the molecular assemblies of the different populations in terms of height and diameter or height profiles, as previously described^30^.

### Antibody preparation and immunoprecipitation

Antibodies were prepared by PRIMM srl (Milano, Italy) according to the experimental protocol approved by the Italian Ministry of Health (Decree number 171/2002-A, Law number 116/1992). Anti-Phe assembly antibodies were prepared as already described by Adler-Abramovich^5^ (2012) with modifications. Briefly, Phe solutions (120 mM in ddH_2_O) were kept for 24 h at room temperature until organized assemblies were formed. Rabbits were immunized with Phe assemblies in Freund’s adjuvant solution 7 times subcutaneously at 0 time, after 21 days and then weekly. Two days after the last injection, the animals were sacrificed and the serum obtained was passed on Sepharose-Protein A (GE Healthcare). Purified immunoglobulins, at 1:10 dilution were incubated overnight at 4 °C with Phe (20 mM) in culture medium to immunoprecipitate Phe assemblies. Moreover, to achieve the complete elimination from the culture medium of the immune complex formed by Phe and the anti-Phe antibody, Sepharose-Protein A beads were added to a distinct batch and kept for 1 h at 4 °C. After centrifugation at 16000 x *g* for 10 min, the supernatant fraction was collected and used for cell treatment.

### Culture and treatment of cell lines

Human neuroblastoma SH-SY5Y, human adenocarcinoma HeLa and human hepatocarcinoma HepG2 cells were grown in Dulbecco’s Modified Eagle Medium (DMEM Lonza) supplemented with L-glutamine (5 mM, Gibco), antibiotics (penicillin/streptomycin 10000 U, Lonza) and 10% heat-inactivated foetal calf serum (Gibco) at 37 °C and 5% CO_2_ in a humidified atmosphere. Cell lines were seeded (10^4^/100 μL) in 96 well plates and incubated overnight. After attachment, the medium was replaced with Phe (Sigma Aldrich) dissolved and diluted in DMEM with lower FCS (1%) to reduce cell growth. Alanine was used as a negative control (Sigma Aldrich). For the study of the efficacy of Doxy (Sigma Aldrich) on Phe toxicity or the inhibition of phenylalanine hydroxylase (PAH) by α-methyl-Phe (Bachem), Doxy was freshly dissolved in ddH_2_O and immediately mixed with Phe or the inhibitor, respectively.

In all experiments cells were treated for 24 h and then their viability was determined by MTT assay. Tetrazolium solution (20 μL of 5 mg/mL, Sigma Aldrich) was added to each well and incubated for 4 h. The medium was replaced with acidified isopropanol (0.04 M HCl) to dissolve the purple precipitate and the absorbance intensity was measured at 570 nm, using a plate reader (Infinite M200, Tecan). Cell viability was calculated as percent of control group (untreated 100%) and expressed as mean ± standard deviation of 3 wells for each experiment, replicated three or four times. Gentamicin (Gibco) was used as a negative control for antibiotic activity without effect on aggregation and fibrillation.

### Primary neuronal cultures

Procedures involving animals and their care were conducted in conformity with the institutional guidelines at the IRCCS Istituto di Ricerche Farmacologiche “Mario Negri” (Quality management System Certificate—UNI EN ISO 9001:2008–Reg. N.8576-A), in compliance with national (D.lgs. 26/2014; Authorization n. 19/2008-A issued March 6, 2008 by Ministry of Health) and international laws and policies (EEC Council Directive 2010/63/UE; US NIH Guide for care and use of laboratory animals, 2011). The preparation of primary neurons from mice was done according to experimental protocols that have been reviewed and approved by the Mario Negri Institute Research Animal Care and Use Committee, project number: 7/03-C, issued on May 2013, according to the Italian Law number 116/1992.

Primary cultures were obtained from the hippocampus, cerebellum and cortex of 13-day-old (E13) C57BL/6J mouse embryos as previously reported^31^. Tissues of interest were dissected and disrupted mechanically (by vigorous pipetting) and enzymatically (exposure to DNAse and trypsin; Sigma Aldrich), before being centrifuged through a BSA cushion. Cells obtained at this step include mixed neuron/glia population.

#### Hippocampal, cerebellar, and cortical neuron cultures

Cells obtained from the hippocampus, cerebellum or cortex were plated at a density of 300,000 cells/cm^2^ into 96-well plates, previously pre-coated with poly-L-lysine (Sigma Aldrich), and maintained in complete culture medium (CCM) containing: Neurobasal, 2% B27, 2% horse serum (Life Technologies), 0.5 mM L-glutamine, 25 μM 2-mercaptoethanol, 1% antibiotics (penicillin/streptomycin; Sigma Aldrich), and 10 ng/mL BDNF (Amgen). Glutamate (25 μM; Sigma Aldrich) was added to the cultures until the 4^th^ day *in vitro* (div). To obtain purified neurons, mixed cultures were treated with 5 μM cytosine arabinoside (Sigma Aldrich) on the second div for 24 h to inhibit the proliferation of glial cells and subsequently maintained in serumless medium.

#### Glial cultures

Glia-enriched cultures were obtained from the cerebral cortices by plating the different cell populations at a density of 25,000 cells/cm^2^ into flasks (pre-coated with poly-L-lysine). Spared neurons were eliminated using culture medium deprived of neurotrophic factors. Culture medium included: DMEM/F-12 (Sigma Aldrich) plus 10% heat-inactivated foetal bovine serum (Euroclone) and 1% penicillin/streptomycin. Purified microglia were obtained by shaking flasks with confluent mixed glial cultures overnight at 275 rpm. Floating cells (mostly microglia) were collected and seeded at a density of 200,000 cells/cm^2^ into 96-well plates for neurotoxicity analysis or on a confluent astrocyte layer (approximately 10% of the astrocyte number) for hippocampal “sandwich” co-cultures. Astrocyte-enriched cultures were obtained by treating the glial cultures, from which microglia had been previously harvested, with 60 mM L-leucine methyl ester (to selectively remove microglia; Sigma Aldrich) for 90 minutes. To prepare a feeder layer for “sandwich” co-cultures, astrocytes were collected and seeded at a density of 25,000 cells/cm^2^ into 12-well plates. For the toxicity analysis, purified astrocytes were seeded at a density of 200,000 cells/cm^2^ into 96-well plates.

#### Hippocampal “sandwich” co-cultures

Low-density purified neurons with healthy morphology and good differentiation were obtained by establishing neuron/glia co-cultures in which the two cell types were not in contact but shared a common medium (CCM), as previously reported^31^. The hippocampal cell suspension obtained from the dissociation of embryo brain tissues was plated at a density of 20,000 cells/cm^2^ on glass coverslips with paraffin dots and maintained in serumless medium. On the second div, coverslips with cultured neuron were transferred into 12-well plates containing the astrocyte feeder layer. The coverslips were inverted so the paraffin dots created a narrow gap that prevented the contact of the two cell types; however, they shared a common medium. The day before treatment, microglia were added (5,000 cells/cm^2^) to the cultures.

#### Culture Treatments

The different cell cultures were exposed to Phe (diluted to the appropriate concentrations, 0.12–60 mM) in culture medium. Cultures maintained with normal medium represented the control condition and alanine solutions were used as negative controls. Cell viability and neuronal alterations were assessed after incubation for 72 h. The effects of Doxy were evaluated after co-treatment with Phe.

### Immunocytochemistry

After treatments, cells were fixed with 4% paraformaldehyde and permeabilised by 0.2% Triton X-100 (Sigma Aldrich). Staining was carried out by overnight incubation with the primary antibodies, followed by the use of appropriate fluorescent secondary antibodies (Dy-light; Rockland Immunochemicals). Cell nuclei were labelled with Hoechst 33258 (Sigma Aldrich) after an incubation with 250 ng/mL solution. Primary antibodies were anti-neurofilament 200 (NF200, rabbit, 1:500; Sigma Aldrich) and anti-synaptophysin (mouse, 1:500; Santa Cruz Biotechnology). Fluorescent secondary antibodies conjugated to different fluorochromes were used at 1:1,000 dilution (goat anti-rabbit tagged with 549 fluorescent dye and donkey anti-mouse tagged with 488 fluorescent dye respectively to recognize NF200 and synaptophysin; Rockland Inc.).

### Cell viability and neuronal alterations assessment

For cell viability assessment, purified hippocampal, cerebellar or cortical neurons, glial cells (microglia or astrocytes) or mixed neuron/glia cultures were treated at the 3^rd^ div for 72 h. Cell viability was assessed by evaluating the metabolic activity with the CellTiter96 non-radioactive cell proliferation assay (MTS test, Promega) following the manufacturer’s instructions.

Neurotoxic alterations (synaptic protein expression and dendritic arborisation) were analysed after immunocytochemistry in hippocampal “sandwich” co-cultures treated at the 10^th^ div for 72 h as reported in Mariani *et al.*^31^ (2015). Z-stack pictures of stained cells were acquired at 600x or 1200x magnification with a laser scanning microscope (Olympus Fluoview BX61 with a FV500 confocal system). Deconvolution, 3D reconstructions and data analysis were performed with the Imaris v7.2 software (Bitplane Inc.). The dendritic arborisations were evaluated via automated Sholl analysis and count of dendritic branches for each NF200-stained neuron. For synaptic analysis, data were expressed as the density of synaptophysin-positive spots normalized on the total volume of neurofilament (revealed with NF200).

### Statistical analysis

In the experiments with cellular lines, data were expressed as the mean with standard deviation and were compared via one-way ANOVA, followed by the appropriate post hoc test (GraphPad Prism). Statistical significances are reported in the figure legends. In the experiments with primary cultures, data were analysed using Student’s *t*-test, one-way ANOVA and the Dunnett’s or Tukey’s tests, or two-way ANOVA and the Bonferroni post-test, using GraphPad v6.01 (GraphPad Software Inc.). The limit of statistical significance was set at *p* < 0.05. The EC50 (effect concentration 50, concentration inducing 50% cell mortality) were calculated using GraphPad Prism software (logarithmic transformation of X-values and nonlinear regression, sigmoidal dose-response analysis with variable slope, with bottom and top constrains set at 0 and 100, respectively). Values are given at ± 95% confidence intervals.

## Additional Information

**How to cite this article**: De Luigi, A. *et al.* Doxycycline hinders phenylalanine fibril assemblies revealing a potential novel therapeutic approach in phenylketonuria. *Sci. Rep.* **5**, 15902; doi: 10.1038/srep15902 (2015).

## Supplementary Material

Supplementary Information
