# Supplementary material for: Doxycycline hinders phenylalanine fibril assemblies revealing a potential novel therapeutic approach in phenylketonuria
Source: Sci Rep. 2015 Oct 29;5:15902. doi: 10.1038/srep15902 (PMC4625134; doi:10.1038/srep15902)
Supplement: Supplementary Information [file srep15902-s1.pdf]

Supplementary Information

Doxycycline hinders phenylalanine fibril assemblies revealing a potential novel therapeutic approach in phenylketonuria.

Ada De Luigi[1], Alessandro Mariani[2], Massimiliano De Paola[2], Andrea Re Depaolini[2], Laura Colombo[1], Luca Russo[1], Valeria Rondelli[3], Paola Brocca[3], Lihi Adler-Abramovich[4], Ehud Gazit[4], Elena Del Favero[3], Laura Cantù[3] and Mario Salmona[1]*.

[1] IRCCS-Mario Negri Institute of Pharmacological Research, Department of Biochemistry and Molecular Pharmacology, Milano, 20156, Italy, [2] IRCCS-Mario Negri Institute of Pharmacological Research, Department of Environmental Health Sciences, Milano, 20156, Italy, [3] Department of Medical Biotechnology and Translational Medicine, University of Milan, LITA, Segrate, 20090, Italy, [4] Department of Molecular Microbiology and Biotechnology, Tel Aviv University, Tel Aviv, 69978, Israel.

*mario.salmona@marionegri.it

Figure S1

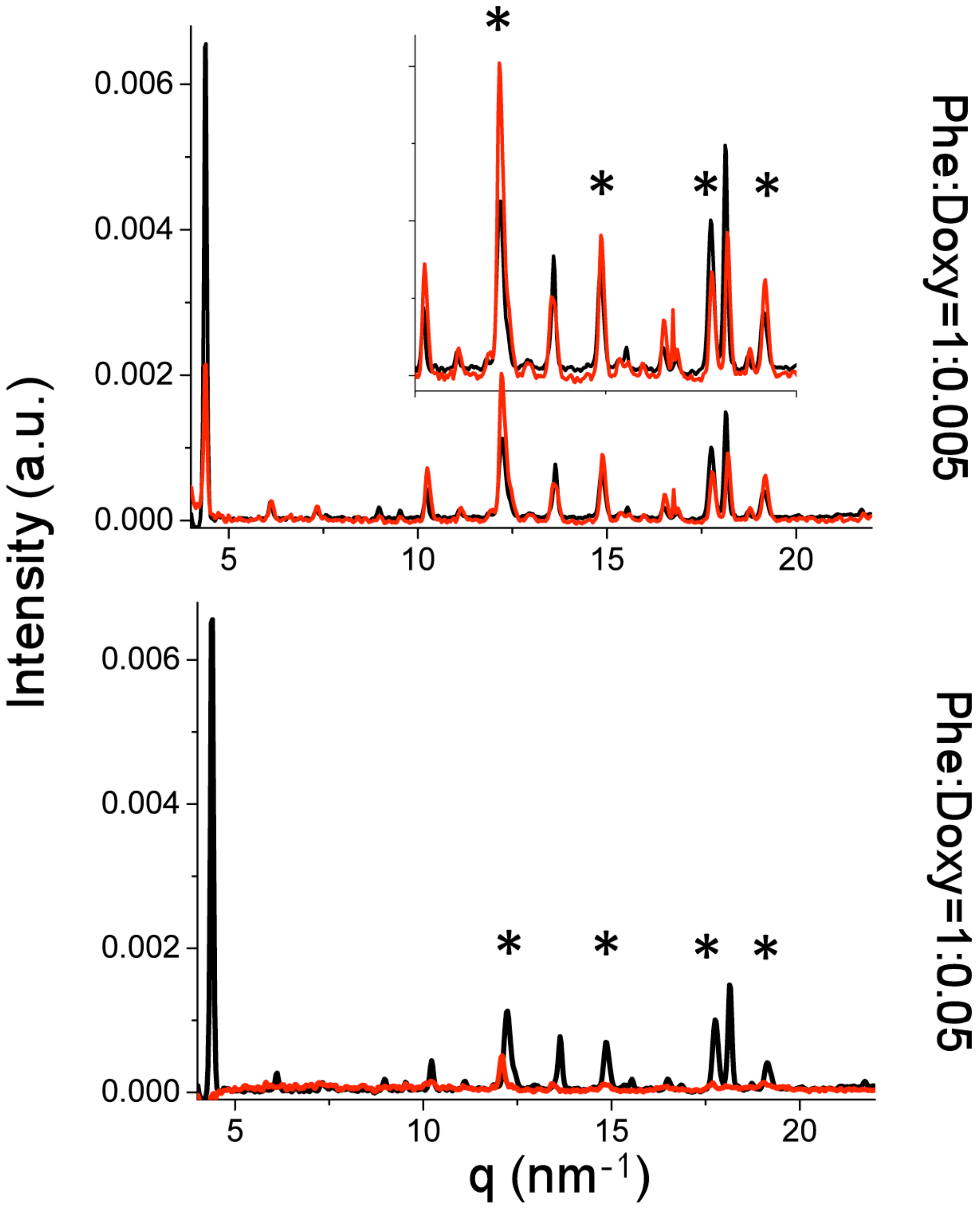

Figure S1. Intensity profiles in the Wide Angle X-ray Scattering region (WAXS) for mixed Phe:Doxy (red line) as compared to pure Phe (black line) for increasing Doxy relative mole ratios. Asterisks mark the equatorial peaks.

Figure S2

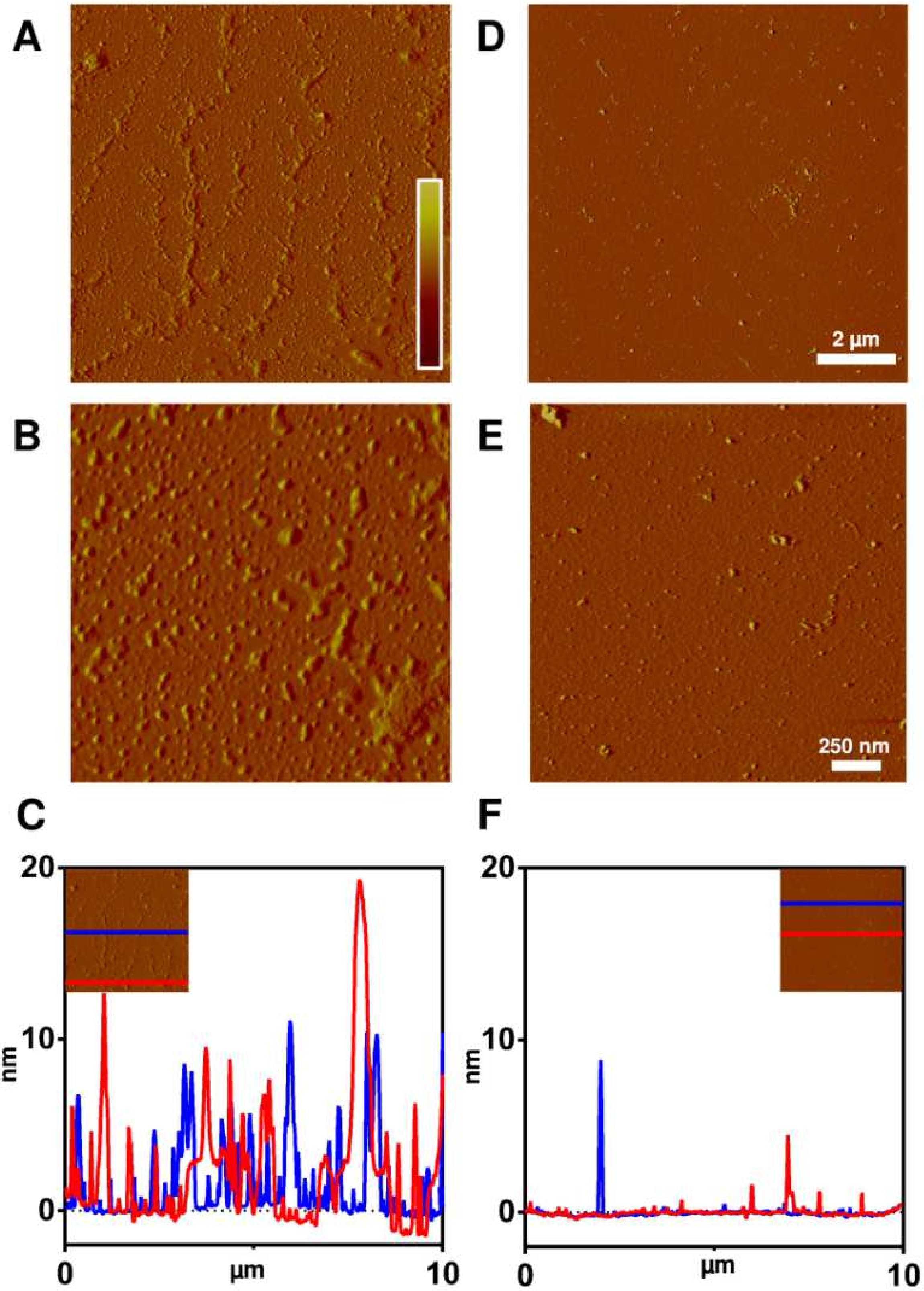

Figure S2. Tapping mode atomic force microscopy images (amplitude data). Images (Panels **A** and **B**) show the assemblies of freshly dissolved 1 mM Phe in 10 mM acetate buffer, pH 4.7 and following co-dissolution with Doxy (Panels **D** and **E**) at 1:0.005 molar ratio. The scale bars correspond to an amplitude range of -100/+80 mV for panel A, -25/+25 for panel B, -20/+20 for panel D and -10/+10 for panel E. Height profiles were determined by SPIP analysis and same colour code was used to represent the regions of images chosen for the measures and their line-profile. Measurement showed a mean height near 10 nm with some peaks reaching 20 nm (Panel **C**).

In presence of Doxy morphologies dramatically changed, with the disappearance of bigger assemblies (Panels **D** and **E**). Measurement showed a decrease of mean height with a major distribution under 3 nm and with no peaks over 10 nm (Panel **F**).

Figure S3

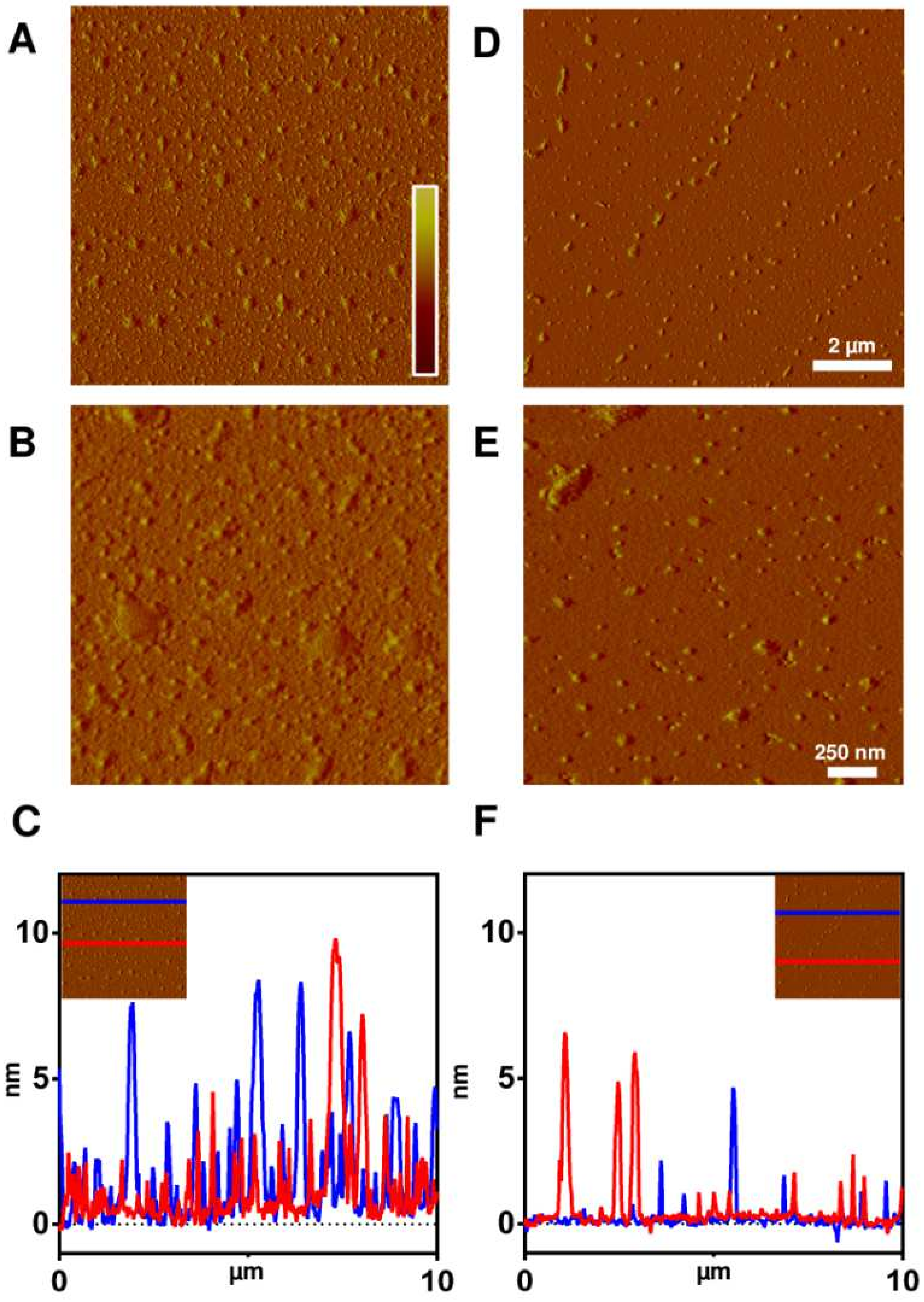

Figure S3. Tapping mode atomic force microscopy images (amplitude data). Images (Panels **A** and **B**) show the assemblies of freshly dissolved 1 mM Phe in 10 mM TRIS-HCl buffer, pH 9.2 and following co-dissolution with Doxy (Panels **D** and **E**) at 1:0.005 molar ratio. The scale bars correspond to an amplitude range of -50/+50 mV for panel A, -25/+20 for panel B, -45/+45 for panel D and -15/+15 for panel E. Height profiles were determined by SPIP analysis and same colour code was used to represent the regions of images chosen for the measures and their line-profile.

Sample containing Phe alone showed a complex morphology with a broad distribution between 6 nm and 10 nm for higher peaks and between 3 nm and 5 nm for the lower peaks (Panel **C**). Co-incubation with Doxy showed a reduction of heterogeneity and complexity, producing structures with height between 5 nm and 2 nm (Panel **F**).
